# Endothelin 1 levels in relation to clinical presentation and outcome of Henoch Schonlein purpura

**DOI:** 10.1186/1471-2431-8-33

**Published:** 2008-09-02

**Authors:** S Fessatou, P Nicolaidou, D Gourgiotis, H Georgouli, K Douros, M Moustaki, A Fretzayas

**Affiliations:** 1 3rd Department of Pediatrics "Attikon" University Hospital, Athens University School of Medicine, Athens, Greece; 2 2nd Department of Pediatrics, "P & A Kyriakou" Children's Hospital Athens University School of Medicine, Athens, Greece; 3 3rd Department of Pediatrics, University of Athens, Attikon University Hospital, 1 Rimini str, Haidari, 12462, Athens, Greece

## Abstract

**Background:**

Henoch Schonlein purpura (HSP) is a common vasculitis of small vessels whereas endothelin-1 (ET-1) is usually reported elevated in vasculities and systematic inflammation. The aim of the present study was to investigate whether ET-1 levels are correlated with the clinical presentation and the outcome of HSP.

**Methods:**

The study sample consisted of thirty consecutive patients with HSP. An equal number of healthy patients of similar age and the same gender were served as controls. The patients' age range was 2–12.6 years with a mean ± SD = 6.3 ± 3 years. All patients had a physical examination with a renal, and an overall clinical score. Blood and urinary biochemistry, immunology investigation, a skin biopsy and ET-1 measurements in blood and urine samples were made at presentation, 1 month later and 1 year after the appearance of HSP. The controls underwent the same investigation with the exception of skin biopsy.

**Results:**

ET-1 levels in plasma and urine did not differ between patients and controls at three distinct time points. Furthermore the ET-1 were not correlated with the clinical score and renal involvement was independent from the ET-1 measurements. However, the urinary ET-1 levels were a significant predictor of the duration of the acute phase of HSP (HR = 0.98, p = 0.032, CI0.96–0.99). The ET-1 levels did not correlate with the duration of renal involvement.

**Conclusion:**

Urinary ET-1 levels are a useful marker for the duration of the acute phase of HSP but not for the length of renal involvement.

## Background

Henoch Schonlein purpura (HSP) is a well known systemic small vessel vasculitis characterized by major manifestations; arthritis, nonthrombocytopenic purpura, abdominal pain and renal disease. The latter is of major concern since it may result in lifelong problems. Although the pathogenesis of HSP remains largely unknown there is strong evidence that IgA has a pivotal role given the increased serum IgA concentrations, IgA with concomitant circulating immune complexes and IgA deposition in vessel walls and renal mesangium have been observed during the course of the disease. Furthermore, activation of the alternative pathway of complement and cytokine abnormalities has also been implicated as having a role in HSP pathogenesis [1].

Endothelins (ETs) were first found by Yanagisawa et al. in 1988 [2] and 3 isoforms of this peptide, ET-1, ET-2, ET-3, have been isolated [3]. Their biological activities cover a wide spectrum which includes regulation of hormones and neurotransmitter, cellular growth and proliferation, bronchoconstriction, natriuresis and water diuresis (4). Urine contains higher concentrations of ET compared to those of plasma which is mainly derived from the in situ production by the kidneys [4].

Endothelin-1 (ET-1) is a potent vasoconstrictor and its concentrations in plasma are increased markedly in a number of pathologies, such as ischemia induced damage and reperfusion, vasculities of various types, congestive heart failure, systemic inflammatory response seen in septic shock syndrome and similar pathology [5]. In a study by Muslu A et al. it was shown that ET-1 plasma levels were significantly higher in HSP patients during the acute phase compared to levels in remission but also to levels in healthy controls [6].

The purpose of this study was to determine whether ET-1 levels in plasma and urine are related to the severity of the clinical presentation and the outcome of HSP.

## Methods

Thirty patients with HSP were recruited during a 2 year period from January 2005 to December 2006. The control group consisted of an equal number of healthy children matched for age and gender. The age range was 2–12.6 years with a mean of 6.3 ± 3 years. The respective ages for the controls were 2–12.7 years and 6.2 ± 2.6 years. Male to female ratio was 14/16 in both groups. Informed consent was obtained from the parents of all participants and the study was approved by the Local Ethics Committee. The diagnosis of HSP was based on the criteria established by the American College of Rheumatology [7]. A punch skin biopsy was performed in all patients in order to verify the diagnosis. Biopsies were obtained from affected and non affected skin within 48 hours of the appearance of the lesions.

A detailed history and a complete physical examination were obtained from all patients. A clinical scoring system adjusted from De Matia D et al. and Muslu A et al. which consisted of the sum of three distinct scores for arthritis, abdominal symptoms and renal involvement were used to assess disease activity and severity [6,8]. This score was modified by using more objective criteria (Table 1). Severity of the disease was determined as mild or severe, if the clinical score was ≤ 4 or > 4, respectively

**Table 1:** The clinical scoring system in patients with HSP (6, 8)

Arthritis score
0 = No symptom
1 = arthralgia and/or slight swelling (normal walk)
2 = arthralgia and/or moderate swelling (limp walk)
3 = arthralgia and/or severe swelling (refuse to walk)

Abdominal score
0 = No symptom
1 = mild abdominal pain(medically elicited) and/or occult blood in stool (+)
2 = moderate abdominal pain (transient complaints brought to medical attention) and/or occult blood in stool (++/+++)
3 = severe abdominal pain and/or melena and/or hematemesis and/or intussusception

Renal score
0 = No proteinuria and/or 3–5 RBC/HPF
1 = proteinuria 30 mg/dl and/or microalbuminuria and/or 10–15 RBC/HPF)
2 = proteinuria 30–150 mg/dl and/or > 50RBC/HPF
3 = proteinuria 150 mg/dl and/or macroscopic haematuria

RBC: red blood cells

Laboratory measurements included complete blood count, erythrocyte sedimentation rate (ESR), C reactive protein (CRP), total protein and albumin, blood and urine electrolytes, immunoglobulins (IgA, IgG, IgM), C3, C4, ASO, ANA, anti-DNA, Ra test, p-ANCA, blood urea nitrogen (BUN), creatinine, culture of pharyngeal swabbing and stool guiac test and routine urinalysis (which was performed every 15 days during the study period). In a random urine specimen taken at the same time with the creatinine blood sample, ET-1(expressed as creatinine ratio) and N-acetyl-b-D glucosaminidase (NAG- expressed as creatinine ratio), was measured as a sensitive marker of tubular damage. Creatinine, total protein and a1 and b2 microglobulin were also calculated in a 24 hours urine collection as tubular markers and microalbumin as a marker of glomerular damage. The children who presented with gross or mild haematuria and/or proteinuria and/or tubular or glomelural involvement were considered as having renal involvement.

Venous blood samples for plasma ET-1 determination were collected in frozen plastic tubes containing EDTA (0.1 ml EDTA/ml of blood) after 30 min of supine rest. They were promptly centrifuged and stored in tubes containing aprotinine at -70°C until analyzed. Plasma ET-1 levels were determined by radioimmunoassay (RIA) (Endothelin 1–21 specific [^125^I] columns (Amersham Biosciences Amprep™ 500 mg C2 columns). The sensitivity of the assay was 1.2 pg/ml. The intra and inter assay variability were 4.8% and 13.8% respectively. Urinary NAG levels were measured following a fluorometric method described by Leadback and Walker [9] as well as by Woolen and Walker [10] and subsequently modified by Gnanadurai et al [11]. The urinary creatinine levels were analyzed by Jaffe's method for calculating NAG/creatinine quotients [12].

Patients were examined and underwent all the aforementioned laboratory tests at 3 distinct time points. The first at the time of diagnosis of HSP corresponding to the acute phase. The second, 1 month later or when renal involvement was diagnosed (which ever happened first) and the third 1 year later which roughly corresponded to the remission of the disease. Four patients continued to present with renal impairment even after 15, 24, 14 and 18 months respectively and for that reason their last examination did not correspond to their remission phase.

### Statistical methods

Continuous variables are expressed as mean ± SD whereas time variables as median (p50), 25^th ^percentile (p25) and 75^th ^percentile (p75). In univariate analysis we compared means between different groups with t-test and one way Anova and we used Spearman's rho to search for any correlations between variables. In multivariate analysis, we used logistic regression to see if ET-1 was a predictive factor for renal damage and Cox Proportional Hazard models which were used to estimate relative risk [expressed as hazard Ratio (HR)] of remission associated with a unit change in a covariate, to explore for any correlations between ET-1 and the length of the disease. We used the backward procedure for selecting the best models. The statistical significance threshold was set at p < 0.05.

## Results

Twenty-three (76.6%) of our patients had mild and 7 (23.4%) had severe disease. Rash was evident in all patients at presentation. In 12 (40%) of them it was preceded by: arthritis, in 6 (20%) by gastrointestinal complaints, in 5 (17%) hematuria in 1 (3%). Histological examination of the skin biopsies revealed typical leukocytoclastic vasculitis in all patients; whereas immunofluorescence studies showed perivascular IgA deposition in 28 (93.3%), C3 deposition in 13(43.3%) and IgM deposition in 5 (16.6%) patients.

During the course of the disease, arthritis occurred in 25 (83%), gastrointestinal involvement in 24 (80%) and renal involvement in 18 (60%) patients. Blood pressure was within normal limits in all patients apart from 1 who developed nephritic syndrome.

Only one patient who finally developed nephrotic syndrome, had low total protein and albumin. Four more patients had proteinuria, which was not at nephrotic levels and 10 microalbuminuria. Levels of NAG, a-1 and b-2 microglobulin were within normal limits in all patients. The clinical hallmark of HSP nephritis is haematuria. In our study 12/30 (40%) patients had microscopic and 5/30 (16.6%) developed gross haematuria. Four patients of the latter group finally developed proteinuria and two of them underwent renal biopsy which revealed glomerulonephritis characterized by diffuse hypercellularity and mesangial proliferation.

Laboratory assays taken at presentation, were within normal limits with the exception of raised IgA in 14/30 (46%), elevated C_3 _values in 1/30 (3.3%) (Table 2), and high ASO titers (> 200 IU/ml) in 17/30 (56%). The culture of pharyngeal swabbing was positive for group A beta hemolytic streptococcus in 11/30 (36%).

**Table 2:** Clinical score and selected laboratory values in patients with HSP, at presentation (IgA, C_3_) and at three distinct time points (ET-1).

					1^st ^time point		2^nd ^time point		3^rd ^time point	
Patient No	Age (years) /Gender (M/F)	IgA (mg/dl)	C3 (mg/dl)	Clinical score	ET-1 in plasma (pg/ml)	ET-1 in urine (pg/ml)	ET-1 in plasma (pg/ml)	ET-1 in urine (pg/ml)	ET-1 in plasma (pg/ml)	ET-1 in urine (pg/ml)
1	5./F	261	204	2	15.388	9.147	16.2	40.112	16.57	39.742
2	9.5/F	162	141	1	17.568	22.843	16.75	60.149	16.78	56.631
3	10/F	802	139	2	9.96	75.957	21.55	24.874	15.64	39.401
4	2/M	101	116	3	13.519	55.413	16.64	25.85	15.89	38.46
5	2/M	107	118	1	9.158	53.148	14.56	23.69	15.6	37.75
6	11.5/M	278	128	3	15.637	23.54	16.63	30.71	16.97	25.589
7	10.5/F	254	141	3	8.099	21.07	14.26	85.07	15.97	61.322
8	4/F	171	108	4	14.329	34.932	17.5	27.258	15.72	45.46
9	5.5/M	323	121	6	18.316	43.31	16.69	52.272	16.47	48.37
10	5.5/M	261	116	7	15.45	40.02	11.4	30.616	11.08	11.505
11	12.5/M	223	172	5	8.659	12.346	10.65	20.706	19.17	113.235
12	2/F	81.7	147	2	14.515	35.614	15.26	42.23	15.97	38.46
13	11/M	218	148	1	16.69	31.3	14.39	22.357	15.9	34.093
14	3.5/F	112	127	1	16.63	34.646	19.62	42.578	23.5	41.68
15	3.5/F	210	104	1	12.46	16.329	17.75	73.666	16.26	45.03
16	8/F	288	137	2	14.57	48.38	17.38	45.133	16.34	44.627
17	8/M	204	151	2	15.64	23.78	17.88	28.657	15.98	36.95
18	8/F	402	116	3	26.53	32.51	18.25	86.823	17.62	89.72
19	5..5/F	133	152	3	17.13	25.918	14.82	30.406	14.98	42.78
20	2/M	129	119	5	13.7	22.774	17.88	52.463	23.45	30.821
21	7/F	239	164	4	16.19	43.74	17.38	38.13	7.78	97.409
22	8.5/M	143	143	2	16.57	20.225	16.01	37.31	15.79	44.63
23	7.5/M	156	106	8	11.21	23.737	18	63.22	23.31	95.465
24	4/F	193	141	3	18.87	16.731	8.72	23.641	15.34	38.538
25	4/M	377	117	4	18.87	33.195	22.75	58.51	19.41	44.75
26	5/M	179	147	3	18.06	48.372	13.39	28.513	8.16	28.113
27	7.5/F	174	139	3	26.8	20.227	21.5	59.855	15.5	19.893
28	6.5/M	284	147	5	19.49	31.143	11.21	51.412	10.77	31.48
29	4/F	114	96	1	12.27	181.192	16.82	25.594	17.55	37.985
30	3.5/F	197	130	7	17.56	41.909	11.58	28.575	14.84	48.859

Normal values of IgA (mg/dl)

4–6 months 4.4–84

7–12 months 11–106

1–5 years 14–159

6–10 years 33–236

Adults 70–312

Normal values of C_3 _(mg/dl)

1–3 months 53–31

3–12 months 62–180

1–10 years 77–195

Adult 83–177

Positive stool guiac test was detected in 16 (53.3%), haematemesis in 2 (6.6%), melena in 4 (13.3%) and intussusception in 1 patient. The duration of time variables expressed as median (P50) and 25^th ^and 75^th ^percentile (p75, p25) in days were 8 (14,5) for the acute phase of the disease, 45(362,5) for microhaematuria and 34(92,5) for microalbuminuria.

No differences were found in ET-1 levels in plasma and urine between patient and control groups at each of the three distinct time points (Table 3). Also there was no correlation between renal or overall clinical score and ET-1 values in plasma and urine on the 3 time points (Table 4, 5) between patients with and without renal involvement (Table 6).

**Table 3:** ET-1 plasma and urine levels at 3 selected time points in HSP patients and controls

		ET-1 in patients (mean ± SD))	ET-1 in controls (mean ± SD)	p
1^st ^measurement	Plasma	15.66 ± 4.30	16.75 ± 5.96	0.41
	Urine	37.44 ± 30.78	45.31 ± 20.33	0.24
2^nd ^measurement	Plasma	16.11 ± 3.26	16.75 ± 5.96	0.60
	Urine	42.01 ± 18.64	45.31 ± 20.33	0.51
3^rd ^measurement	Plasma	16.14 ± 3.60	16.75 ± 5.96	0.63
	Urine	46.95 ± 23.15	45.31 ± 20.33	0.77

**Table 4:** ET-1 values in plasma and urine at three distinct measurements in relation to renal score

		ET-1 (mean ± SD, range)	Renal score (Mean ± SD, range)	rho	p
1^st ^measurement	Plasma	16.20 ± 5.18, 2.18–26.80	0.70 ± 0.91, 0–3	-0.011	0.95
	Urine	41.38 ± 26.17, 9.14–181.19		0.099	0.59
2^nd ^measurement	Plasma	16.11 ± 3.26, 8.72–22.75	0.73 ± 1.08, 0–3	-0.180	0.92
	Urine	42.01 ± 18.64, 20.70–86.82		0.248	0.57
3^rd ^measurement	Plasma	16.14 ± 3.60, 7.78–23.5	0.1 ± 0.30, 0–1	-0.288	0.52
	Urine	46.95 ± 23.15, 11.50–113.23		0.473	0.66

Results from univariate analysis (rho = spearman's correlation coefficient)

**Table 5:** ET-1 values in plasma and urine at three distinct time point measurements in relation to clinical score.

		ET-1 (mean ± SD, range)	Clinical score (mean ± SD, range)	rho	p
1^st ^measurement	Plasma	16.20 ± 5.18, 2.18–26.80	3.23 ± 1.94, 1–8	0.145	0.443
	Urine	41.38 ± 26.17, 9.14–181.19		-0.032	0.864
2^nd ^measurement	Plasma	16.11 ± 3.26, 8.72–22.75	0.73 ± 1.08, 0–3	-0.018	0.923
	Urine	42.01 ± 18.64, 20.70–86.82		-0.106	0.576
3^rd ^measurement	Plasma	16.14 ± 3.60, 7.78–23.5	0.13 ± 0.34, 0–1	0.011	0.952
	Urine	46.95 ± 23.15, 11.50–113.23		0.237	0.205

Results from univariate analysis (rho = spearman's correlation coefficient).

**Table 6:** ET-1 values in plasma and urine between patients with and without renal involvement (RI).

		Patients with RI	Patients without RI	p
1^st ^measurement	Plasma	15.77 ± 4.48, 8.6–26.8	15.56 ± 4.29, 8.09–26.53	0.851
	Urine	35.59 ± 16.80, 12.34–75.95	39.06 ± 39.77, 9.147–181.19	0.506
2^nd ^measurement	Plasma	16.11 ± 3.26, 8.72–22.75	16.20 ± 3.25, 8.72–22.75	0.891
	Urine	38.40 ± 14.64, 20.70–63.22	44.41 ± 20.94, 23.64–86.823	0.582
3^rd ^measurement	Plasma	16.86 ± 4.61, 11.08–23.31	16.00 ± 3.47, 7.78–23.5	0.977
	Urine	60.63 ± 42.53, 11.50–113.23	44.22 ± 17.30, 19.89–97.40	0.486

Of note, in order to investigate if any correlation existed between ET-1 in acute phase and renal involvement, we used two logistic regression models (one for ET-1 in plasma and one for ET-1 in urine) and in each one we adjusted for potential confounders namely, age, gender and clinical and laboratory parameters that were measured during the acute phase of the disease i.e. clinical score, ESR, CRP, IgA and C3. Our results show that renal involvement and ET-1 were not correlated (OR = 1.05, 95% CI 0.79–1.383, p = 0.74 and OR = 0.99 95% CI 0.95–1.03, p = 0.71 for ET-1 in plasma and urine respectively).

Using Cox Proportional Hazards models and controlling for the same potential confounders as above, we tried to investigate whether ET-1 was correlated with the length of the acute phase of the disease. We found that there was no significant correlation with ET-1 in plasma but ET-1 in urine was a significant predictor for the length of the acute phase (HR = 1.01, p = 0.79, 95% CI 0.91–1.11 and HR = 0.98, p = 0.032, 95% CI 0.96–0.99 for ET-1 in plasma and urine respectively). The latter result implies that the higher the levels of ET-1 in urine, the longer the acute phase. A more precise expression of the latter result is that for one unit increase in ET-1 in urine, the mean rate of remission of acute phase (instantaneous remission rate) is reduced about 2%.

We also investigated the relation between ET-1 levels during the acute phase and the length of microhaematuria and microalbuminuria adjusting for the same potential confounders as before. We found that ET-1 in plasma and urine did not correlate with renal involvement although ET-1 in plasma showed a trend toward a significant correlation with the duration of microhaematuria (H.R. = 1.51, p = 0.055, 95% CI 0.99–2.34).

## Discussion

HSP is the most common vasculitis in childhood. It is a multisystem disease most commonly affecting skin, joints, gastrointestinal tract and kidneys although other organs may also be affected [13]. Its pathogenesis remains largely unknown. However, it is generally considered to be an immune complex-mediated disease characterized by the presence of polymeric IgA1 containing immune complexes which are mainly found in dermal, gastrointestinal and glomerular capillaries [14]. IgA aggregates or IgA complexes with complement deposits in target organs, result in elaboration of inflammatory mediators, including vascular prostaglandins such as prostacyclins which are thought to play a role in the pathogenesis of HSP vasculitis [15].

Alterations in the production of interleukins and growth factors may also have a role in the pathogenesis of HSP. Tumor necrosis factor (TNF), interleukin 1(IL-1) and interleukin-6 (IL-6) may mediate the inflammatory process present in HSP [1,16,17]. There is only one study which demonstrated that plasma ET-1 levels in the acute phase were significantly higher compared to the remission phase and to the controls. However plasma ET-1 levels did not correlate with the clinical and laboratory findings with the exception of a minority of patients with severe disease [6]. In our study we did not find any differences of plasma and urine ET-1 levels between patients and controls.

ET was initially described as a peptide released from large vessel endothelial cells [2]. However other sites of synthesis have subsequently been discovered. ET is also expressed in brain, lung, heart and kidney. As far as the latter location is concerned endothelial, glomerular, tubular epithelial and mesangial cells of the kidney can all synthesize ET [18].

The circulating half life of ET is short (2–3 min) and it is almost completely metabolized in the pulmonary capillaries by the enzyme neutral endopeptidase. A small amount of circulating ET is cleared in the urine [19]. Depending on the nature and the magnitude of the stimulus circulating ET may be elevated in different pathophysiological or experimental conditions. However it is not clear whether an elevated level of circulating ET is necessary for its actions [20]. The effects of ET are detected long after the normalization of the plasma level. These data suggest that the concentration of plasma ET does not correlate with its activity in different cells and tissues and the circulating levels depend on the balance of the luminal endothelial synthesis and metabolism [4].

The kidney and particularly the renal medulla expresses the highest concentration of ET-1 receptors of any organ and also synthesizes ET-1 [21]. Glomerular epithelial and mesangial cells, renal tubular cells and medullary collecting duct cells synthesize and release ET-1. ET-1 is a major mediator of renal vascular tone, tubular secretion of electrolytes and water and vascular smooth muscle and mesangial cell proliferation [22]. ET-1 maintains normal kidney perfusion by increasing renal blood flow, effecting on natriuresis and diuresis [23].

Despite the higher concentration of ET in urine compared to plasma, the urinary excretion of ET does not correlate with the glomerular filtration rate filtered load or plasma levels [24]. It has been suggested that the filtered ET is subject to proteolytic degradation by neutral endopeptidase across the brush border of the proximal tubuli [19]. ET can be a marker of renal injury in different pathological processes in children and in adults but its specificity is low. Therefore ET should be used with caution as a marker of distal tubular or collecting duct epithelial function [4]. ET/creatinine ratio in random urine samples could be used as a reliable index of urinary excretion of ET [25,26]. In our study this urinary ET-1 was correlated with the duration of the acute phase of the disease.

Several systemic rheumatic diseases in which vascular pathology is related to endothelial cell activation appear to be causally related to endothelin.

They include systemic sclerosis, systemic lupus erythematosus, Takayasu and giant cell arteritis, Raynaud's phenomenon, mixed cryoglobulinaemia, aortoarteritis and Buerger's, Bechcet, Kawasaki, mixed connective tissue and Chaga's disease [27-38]. All these studies measured only plasma ET-1 levels in contrast to our study which measured plasma and urine ET-1 levels (although no differences between patients and controls were found).

## Conclusion

In conclusion, plasma ET-1 levels cannot be utilized as a predictor of HSP duration or severity whereas urine ET-1 levels are correlated with the duration of the acute phase of the disease.

## Competing interests

The authors declare that they have no competing interests.

## Authors' contributions

SF and PN had substantial contributions to conception and design and analysis and interpretation of data and drafting of the manuscript. DG and HG made all the necessary biochemical measurements MM and AF have been involved in drafting and revising the manuscript.  KD performed the statistical analysis of the data

## Pre-publication history

The pre-publication history for this paper can be accessed here:


